# Hepatic steatosis in hepatitis C is a storage disease due to HCV interaction with microsomal triglyceride transfer protein (MTP)

**DOI:** 10.1186/1743-7075-7-13

**Published:** 2010-02-23

**Authors:** Silvia Mirandola, David Bowman, Mahmood M Hussain, Alfredo Alberti

**Affiliations:** 1 VIMM-Venetian Institute of Molecular Medicine, Padova, Italy; 2 Department of Cell Biology and Pediatrics, SUNY Downstate Medical Center, Brooklyn, New York, USA

## Abstract

Liver steatosis is a frequent histological feature in patients chronically infected with hepatitis C virus (HCV). The relationship between HCV and hepatic steatosis seems to be the result of both epigenetic and genetic factors. *In vivo *and *in vitro *studies have shown that HCV can alter intrahepatic lipid metabolism by affecting lipid synthesis, oxidative stress, lipid peroxidation, insulin resistance and the assembly and secretion of VLDL. Many studies suggest that HCV-related steatosis might be the result of a direct interaction between the virus and MTP. It has been demonstrated that MTP is critical for the secretion of HCV particles and that inhibition of its lipid transfer activity reduces HCV production. However, higher degrees of hepatic steatosis were found in chronic hepatitis C patients carrying the T allele of MTP -493G/T polymorphism that seems to be associated with increased MTP transcription. We propose here that liver steatosis in hepatitis C could be a storage disease induced by the effects of the virus and of its proteins on the intracellular lipid machinery and on MTP. Available data support the hypothesis that HCV may modulate MTP expression and activity through a number of mechanisms such as inhibition of its activity and transcriptional control. Initial up regulation could favour propagation of HCV while down regulation in chronic phase could cause impairment of triglyceride secretion and excessive lipid accumulation, with abnormal lipid droplets facilitating the "storage" of virus particles for persistent infection.

## Introduction

Hepatic steatosis, defined as excessive lipid accumulation in the cytoplasm of hepatocytes, is a frequent histological feature in patients with chronic hepatitis C (CHC) infection [1-3]. Histological examinations show that up to 50% of these patients have variable degrees of hepatic steatosis [4], even in the absence of other possible steatogenic factors, like alcohol, drugs or metabolic syndrome [5]. Early electron microscopy studies conducted in experimentally infected chimps or parenterally infected humans with non A and non B hepatitis showed presence of abnormal cytoplasmic vesicular changes [6]. In hepatitis C Virus (HCV) infected patients liver steatosis is mainly macrovesicular [7] and is located in the periportal area rather than in the centrilobular area [8], in contrast to what is observed in non-alcoholic fatty liver disease (NAFLD) and in alcoholic liver disease. Prevalence of liver steatosis in HCV patients is significantly higher when compared to patients with other forms of chronic liver disease such as hepatitis B or autoimmune hepatitis, suggesting a direct effect of HCV replication in the development of excess fat accumulation in the liver [9-11]. This is also supported by the observation that the degree of liver steatosis is directly related to the level of HCV replication as measured by serum HCV RNA, at least in patients with HCV-3 infection, in the absence of confounding metabolic causes of steatosis [12,13]. Understanding mechanisms that cause hepatic steatosis in the HCV infected patients has been made difficult due to the co-existence of several confounding metabolic cofactors. Patients with CHC may develop hepatic steatosis as a consequence of concomitant metabolic syndrome, possibly associated with type 2 diabetes, obesity or increased body mass index (BMI). These conditions are quite frequently observed in HCV patients and may cause variable degrees of hepatic steatosis by mechanisms that are similar to those of classical NAFLD, mainly through insulin resistance [12,13]. Indeed, two main types of steatosis have been proposed to coexist in patients with hepatitis C. The first is a *metabolic *type of steatosis that is seen mainly in HCV-1 infected patients and is associated with increased BMI, hyperlipidemia, and insulin resistance. The second is a *viral type *of steatosis that develops also in the absence of any other steatogenic cofactors and that seems to be directly triggered by the virus [14].

Furthermore, HCV may also be involved as a cofactor in the development of the metabolic type of steatosis, as HCV itself was shown to induce insulin resistance that consequently may favour the development of hepatic steatosis [15-17]. Alternatively, HCV may directly affect genes involved in lipid metabolism leading to fat accumulation in the liver. A direct mechanism is likely to prevail in patients who develop extensive steatosis in the absence of insulin resistance as typically, but not exclusively, seen in HCV-3 infected patients. Several lines of evidence indicate a direct correlation between HCV-3 infection and liver fat accumulation. It is known that steatosis resolves after reduced virological response when achieved through antiviral therapy with interferon-alpha and/or ribavirin [18]. We and others have demonstrated that chronic HCV-3 infection correlates with lower serum levels of cholesterol, triglyceride and apolipoprotein B (apoB) compared to patients chronically infected with other HCV genotypes, suggesting a profound alteration in lipid and lipoprotein metabolism in infected hepatocytes [13,19]. Several mechanisms have been proposed to explain how HCV infection may induce liver steatosis. In this review, we propose that HCV-related steatosis might be a viral storage disease due to a specific interference of HCV with intrahepatic lipid metabolism involving hepatic microsomal triglyceride transfer protein (MTP) [19].

## HCV and Hepatic Steatosis

**Figure 1:**
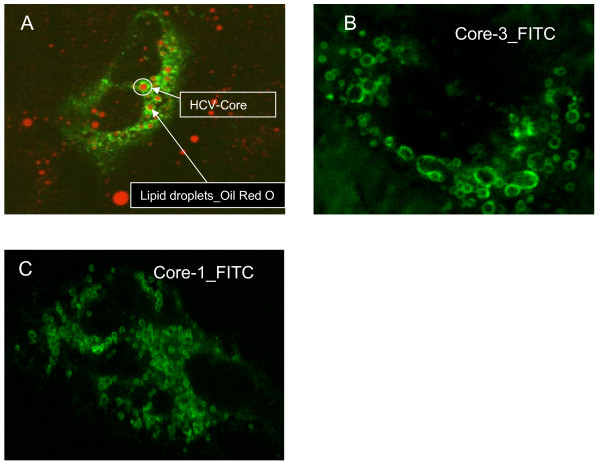
**Localization of HCV Core protein to lipid droplets**. Huh-7 cells were transfected with a plasmid expressing the capsid (Core) protein from hepatitis C virus genotype 1 or genotype 3. Following transfection, cells were fixed, neutral lipids were stained by Red-Oil and core protein was localized by immunofluorescence using an anti-core primary and FITC-conjugated secondary and visualized on a confocal microscopy: 1A) Merged image demonstrating colocalization of HCV core protein (green) and neutral lipids (red) as intracellular lipid droplets 1B) FITC localized genotype 3 core protein and 1C) genotype 1.

The current understanding of the mechanisms leading to lipid accumulation in the cytoplasm of HCV-3 infected hepatocytes is limited, as most of the reported studies used HCV genotype 1 surrogate systems which have not been shown to recapitulate the steatogenic effect *in vivo*. Further encumbering investigators is the lack of robust cell culture systems or animal models for HCV. However, there is strong evidence to suggest that some HCV proteins, particularly the structural capsid protein, core, and the non-structural protein, NS5A, can induce hepatic steatosis by interfering with intracellular lipid metabolism [20-26]. Hepatoma cell lines over-expressing HCV core proteins show increased lipid accumulation. Abid et al. [27] found that lipid staining of transfected cells was significantly higher than that of non-transfected cells for all HCV genotypes tested. However, the accumulation of triglycerides and cytoplasmic lipid droplets was most pronounced with genotype 3a compared to other genotypes. Similar results were more recently reported by Piodi et al. [28], describing that the core protein of both HCV genotypes 1b and 3a has a subcellular localization on the surface of lipid droplets mainly in a macrovesicular pattern. Neutral lipid accumulation was increased if cells were transfected with core protein of genotype 3a. This finding is confirmed by our experiments in hepatoma cell lines (HuH-7) transfected with core proteins as shown in Figure 1. However, as another important finding of this study [28], core protein seems necessary but not sufficient for inducing steatosis, as there were no genetic or functional differences between genotype 3a core proteins from patients with and without HCV-induced steatosis. These data suggest that interactions between HCV core protein and lipid droplets could contribute to steatosis. However, it must be inferred that other host factors and viral proteins are most likely required in the development of HCV related steatosis.

Several mechanisms by which HCV and its proteins might cause excessive lipid accumulation have been suggested and widely discussed in many reviews. All of the proposed hypotheses focus on complex interactions between HCV proteins, lipid synthesis, oxidative stress, lipid peroxidation, insulin resistance and the assembly and secretion of apoB-lipoproteins, all of which might ultimately contribute to the onset of steatosis [29-36]. However, there has been no discussion whether steatosis might be advantageous for the virus. Why HCV might want to induce steatosis?

## HCV, lipoproteins, and microsomal triglyceride transfer protein

In infected patients, HCV particles circulate as low-density lipoprotein (LDL)-virus complexes rich in triglycerides. These so-called lipo-viral particles (LVPs) were found to contain viral RNA, the viral structural proteins, core and envelope glycoproteins E1 and E2, but surprisingly also host-derived apolipoproteins B and E (apoB and apoE) [37], which are the components of apoB-lipoproteins [chylomicrons, very low density lipoproteins (VLDL) and low density lipoproteins (LDL)] [38]. The reasons for the circulation of the virus with lipoproteins are not clear. It is possible that this allows the virus to avoid recognition by leukocytes and also provides a mechanism to enter cells as a surrogate along with lipoproteins.

There is strong evidence for an association between viral infection and lipoprotein metabolism. In cell culture experiments, HCV subgenomic replicons have been shown to affect lipoprotein secretion by interfering with the formation of secretion-competent apoB lipoproteins via inhibition of microsomal triglyceride transfer protein (MTP), an essential chaperone for the biosynthesis of these lipoproteins [39]. Moreover, apoB seemed to interact with the HCV non-structural protein NS5A, suggesting that apoB may be a target of HCV [40]. Recently, membrane vesicles containing the HCV replication complex from Huh7 cells that harbour HCV replicons were isolated [41]. Proteomic analysis of these vesicles revealed that they were enriched in apoB, apoE, and MTP, proteins known to associate with lipoproteins [41]. Interestingly, VLDL synthesis is not required for HCV RNA replication as HCV RNA can replicate in HeLa and HEK-293 cells [42,43], which do not produce VLDL. The reasons for the co-localization of the HCV replication and proteins involved in lipoprotein metabolism have not yet been elucidated. But, mounting evidence suggests a requirement for co-assembly or secretion of VLDL and HCV particles. Using MTP inhibitors and siRNA targeting apoB in a cell culture system constitutively producing infectious hepatitis C virus, Huang et al. [41] demonstrated a dependence on the assembly and secretion of VLDL for HCV production. While the MTP inhibitor significantly decreased both extracellular HCV RNA and infectivity, intracellular RNA levels were unaffected, suggesting the effect was not due to reduced viral replication, but rather due to concomitant disruptions of VLDL assembly and secretion with viral assembly and egress. These results were further confirmed in another study where authors demonstrated that infectious HCV particles assembly and secretion is a highly regulated system in which apoB is a rate-limiting factor and that these two steps require active MTP as shown by the evidence that MTP inhibition reduces the production of infectious intracellular HCV and its secretion into the extracellular milieu [44]. More recent experiments [45] have shown that the secretion of HCV envelope glycoproteins, in the absence of other viral proteins, also seems to require the machinery of apoB lipoprotein assembly. Taken together, these data suggest for MTP-dependent co-assembly of hepatitis C virions with apoB-containing triglyceride rich lipoproteins.

The clinical evidence that HCV infected patients with severe steatosis have reduced serum levels of cholesterol and of apoB is consistent with the hypothesis of a possible involvement of MTP. The role of MTP in the development of HCV-related steatosis has been investigated using different approaches involving animal models, *in vitro *cellular studies and human studies in which others and we have evaluated MTP genetic variability and gene and protein expression in HCV patients. The pivotal observations came from experiments in mouse models: Shintany et al reported that HCV core transgenic mice develop insulin resistance associated with massive hepatic steatosis [46]. Using a similar model, Perlemuter et al. demonstrated that HCV core protein induces hepatic fat accumulation in mice by inhibiting MTP activity, leading to impaired secretion of VLDL [47]. The first study in HCV infected patients was conducted in our laboratory at the Venetian Institute of Molecular Medicine where we investigated MTP gene expression and protein activity in liver biopsy specimens from a series of untreated patients with CHC [19]. Our data indicated that hepatic MTP gene expression and protein activity were reduced in the presence of severe steatosis, particularly in patients infected with HCV-3 and that in these same cases, impairment in MTP activity showed a direct relation to levels of HCV replication. We also observed reduced serum levels of cholesterol and VLDL in patients with impaired MTP expression and severe steatosis in hepatocytes. Our results were confirmed in a more recent study by McPhersons et al [48] who also demonstrated reduced hepatic expression of MTP mRNA in HCV infected patients when compared to a control group of HCV-negative subjects and an inverse relationship between MTP mRNA levels and degree of liver steatosis. Moreover, in this study, an inverse relationship was found also between HCV related steatosis and liver expression of SREBP-1c and GPAT, two genes involved in fatty acid and triglyceride synthesis. These findings support the hypothesis that HCV may cause accumulation of lipids in infected hepatocytes by perturbing hepatic lipid metabolism through a direct interference with MTP, leading to VLDL/triglyceride retention. It remains to be determined whether this lipid retention involves storage of apoB-containing lipoprotein particles due to abnormal assembly and secretion or increased assimilation of cytosolic lipid droplets devoid of apoB.

## MTP Genetic polymorphism and HCV-related liver steatosis

Apart from viral and metabolic factors, some specific host genetic polymorphisms may also play a role in the pathogenesis of steatosis. Genetic polymorphisms can modulate the concentration of MTP protein in the endoplasmic reticulum, which may have an impact on the secretion pattern of lipoproteins. A common polymorphism in the promoter region of the MTP gene, -493G/T, has been characterized functionally. The T allele associates with increased MTP transcription *in vitro *and low serum levels of low-density lipoprotein (LDL) cholesterol in healthy subjects [49]. The MTP -493G/T polymorphism has been implicated in the susceptibility to develop steatohepatitis in patients with type II diabetes [50]. The G allele was more frequently found in patients with non-alcoholic steatohepatitis (NASH) compared with healthy controls, and NASH patients with the homozygous genotype GG showed more severe degrees of liver steatosis [51]. More recently, the role of MTP polymorphism has been investigated in patients chronically infected with HCV. Initially, the MTP -493G/T polymorphism was examined among a set of eight genes that have been reported to have an association with hepatic fibrosis in a group of 326 patients with CHC [52]. Homozygosity of either the G or the T allele revealed an adjusted odds ratio of 4.1 associated with a more rapid progression to liver fibrosis. The association between MTP variants and hepatic steatosis was not tested in this study. Petit et al. assessed the association between the MTP -493G/T polymorphism and steatosis in HCV infected patients for the first time but no relationship was found [53]. However, this study included a cohort of only 86 HCV-positive subjects, with only 39 demonstrating signs of steatosis. Because of the small sample size, subgroups could not be analysed separately, and the analysis of data was done on the whole population and not according to HCV genotypes. More recently Zampino et al analysed 102 patients with CHC [54]. Patients infected with HCV-3 and carriers of the MTP T allele showed higher degrees of steatosis, higher serum levels of HCV RNA and more advanced fibrosis. These patients, irrespective of MTP genotype, had lower serum levels of cholesterol, apoB, HDL and LDL. No such associations of MTP variants were seen in patients infected with HCV genotypes other than HCV-3, probably due to the small sample size of HCV-1 infected patients, generating a type II error (false negative).

We recently evaluated the role of MTP -493 G/T polymorphism in a larger cohort of 298 patients with CHC [55]. Our results confirm a significant association between MTP polymorphism and liver fat accumulation. Age, BMI, HCV-3 and MTP T allele were independent risk factors for high grades of steatosis in the total cohort of HCV patients. In HCV genotype non-3 patients, the MTP T allele was the strongest predictor for severe steatosis. Thus, severe liver steatosis in HCV patients correlates with the MTP T allele, in contrast with what is seen in patients with metabolic syndrome or type II diabetes [50] or NASH [51], in whom genotype MTP -493 GG and the G allele were associated with more severe steatosis.

**Figure 2:**
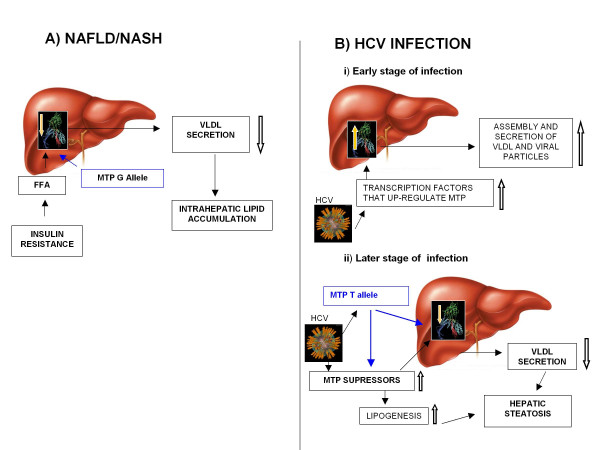
**Possible mechanisms of non-viral and viral steatosis**. (A) In NAFLD/NASH, subjects are insulin resistant and have high plasma free fatty acids (FFA). High FFA delivery to the liver enhances lipoprotein production. Presence of MTP -493G allele, which associates with lower MTP transcription, may lead to impairments of VLDL secretion and consequently to severe intrahepatic lipid accumulation. (B) In HCV infected individuals MTP mRNA and protein levels could be a consequence of up/down-regulation of either suppressors or activators of MTP expression by HCV. During early stages of infection MTP transcriptional activity might be enhanced to facilitate assembly and secretion of infectious HCV-particles (i). At a later stage of infection (ii), HCV may decrease MTP expression through an up-regulation of some MTP suppressors. This suppressor might decrease MTP expression. In addition, this suppressor may increase lipogenesis leading to lipid accumulation and hepatic steatosis. In the presence of T allele at -493 site, decrease in MTP mRNA levels may occur either through a direct binding of some HCV proteins at the -493 site or through an up-regulation of MTP-suppressor(s) by HCV thus contrasting the enhancing effect of the T allele on MTP gene expression. We speculate that increased lipid droplets accumulation in hepatocytes may provide a safe environment for HCV latency.

These differences can be explained by the different mechanisms underlying liver fat accumulation in the two conditions. In patients developing NAFLD/NASH due to metabolic syndrome/type 2 diabetes, insulin resistance is associated with increased free fatty acid delivery to the liver (Figure 2A). Therefore, presence of MTP -493 G allele, which associates with decreased MTP transcription, may lead to more severe impairment of VLDL assembly and secretion, with higher grades of hepatic steatosis. Thus, in these patients liver steatosis is a consequence of liver overload by circulating lipids that can be less efficiently processed by hepatocytes in the presence of lower MTP activity with the G allele. But, why should steatosis be associated with decreased MTP and with T allele in chronic HCV infected patients?

## Complex relationship between MTP and HCV

The studies summarized above indicate a complex relationship between MTP and HCV. First, MTP is essential for the assembly and secretion of HCV. Second, higher MTP activity is conducive for infected cells to secrete HCV-particles. Therefore, intuition predicts that HCV should up-regulate MTP expression to facilitate its propagation. This hypothesis has been confirmed by a recent study [56] in which authors examined the mRNA levels of a set of lipid metabolism-associated genes in the liver of HCV-1 infected patients irrespective to histological features. Interestingly, it was shown that expression levels of MTP and other cholesterol-associated genes were higher in HCV subjects compared to control HCV-negative individuals. Therefore, we propose that HCV might "modulate" transcription of MTP to facilitate synthesis and secretion of infectious particles. It is known that MTP transcription is modulated by different transcription factors that directly bind to the 5'-flanking 150 bp of the MTP gene promoter [57-60]. This region contains a negative regulatory region that includes binding sites for insulin (-124/-116) and sterol response element binding protein (SREBP) (-122/-111). The proximal promoter also contains several positive regulatory HNF1α (HNF1), LRH-1, and HNF4α (HNF4) binding sites. In addition, a regulatory DR1 element that binds to PPARα/RXRα, FOXA2 or COUP-TFII [57,59-62] has been identified. Except for the binding of COUP-TFII that acts as a repressor, most of the transcription factors that bind to DR1 element act as activators [63]. Many studies have shown that several transcription factors involved in MTP transcription are modulated by HCV (Table 1) supporting the hypothesis that changes in MTP mRNA and protein levels could be a consequence of transcriptional regulation during HCV infection.

**Table 1:** Modulations induced by HCV on the transcription factors involved in the regulation of MTP expression

Study	Transcription factor	Effect of HCV
Waris G et al. [69]	SREBP-precursor (Sterol response element binding protein)	HCV-2 infection leads to activation of three isoforms of SREBPs (forms 1, 1c and 2). Moreover NS4B and core protein of genotype 3 activates SREBPs through proteolytic cleavage.

Qadri I et al. [70]	HNF1 and HNF4 (hepatocyte nuclear factor)	Increased expression of HNF1 and HFN4 mRNA in HCV subgenomic replicon-expressing Huh.8 cells. The ability of HCV to induce HNF1 and HNF4 is attributed to 1) increased oxidative stress and 2) direct protein-protein interactions between HCV non-structural component (NS) 5A and HNF1, leading to enhanced HNF1 DNA binding.

Yamaguchi A et al. [71]	PPAR-α (peroxisome proliferator-activated receptor)	In HCV core protein-expressing mice PPAR-α was down-regulated

Kim KH et al. [72]	PPAR-γ	The NS5A increases the transcriptional activity and gene expression of PPARγ

Tsutsumi T et al. [73] Yamaguchi A et al [71]	RXRα (retinoid X receptor alpha)	HCV-Core binds to and activates RXRα

Tanaka N et al. [74]	PPAR-α,	Persistent PPARα activation is essential for development of severe hepatic steatosis and its progression into hepatocellular carcinoma in the liver of core gene transgenic mice

Dharancy et al. [75]	PPAR-α,	Reduced mRNA levels of PPAR-α in HCV infected patients and in HCV core-expressing HepG2 cells

However, under chronic HCV infection, MTP mRNA and activity levels decrease [19]. Higher concentration of HCV proteins might suppress MTP transcription as well as directly inhibit MTP activity contributing to steatosis. As discussed before, during early stages of HCV infection MTP transcriptional activity might be enhanced to facilitate assembly and secretion of infectious HCV-particles. Subsequently, virus might undergo intracellular "storage" in cells for long-term latency. During this process, virus might up-regulate lipogenesis and down regulate as well as inhibit MTP. These processes would lead to significant accumulation of lipids (steatosis) in hepatocytes. Virus could then remain associated with these lipid droplets. Hence, these lipid droplets may provide a safe heaven for its retention and persistence (Figure 2B).

Furthermore, mechanism(s) underlying higher degrees of steatosis in the presence of MTP T allele need clarifications. It is possible that the presence of T allele in the -493 site might favour an interaction with some HCV proteins which in turn modulates MTP transcription. This hypothesis is derived from evidence that specific binding to the MTP -493 site by nuclear proteins decreased transcriptional activity of the MTP promoter [49]. Although in our previous study [55] MTP genotypes did not significantly modify the corresponding mRNA expression, it should be noted that with the TT genotype MTP expression was lower than expected favouring the hypothesis that a negative regulation occurs. It is also possible that chronic phase of HCV infection the presence of T allele in the -493 site may favour the up-regulation of some MTP suppressors by HCV thus contrasting the enhancing effect of the T allele on MTP gene expression.

## MTP: A possible new target for the treatment of HCV infection?

Despite current advances in treatment options, more effective and safer antiviral agents for hepatitis C are clearly needed. About 40% of people who are infected with the hepatitis C virus (HCV) worldwide do not respond to long-term treatments with the best available current modality (combination of peg-interferon and ribavirin) in spite of full compliance with dosing and duration of therapy. Clearly, new therapies are needed to obliterate this global disease. Potentially effective, novel therapeutic strategies could exploit the reliance on and association of the virus with the machinery of host lipid metabolism. Several recent studies and reviews have indeed addressed such therapeutic targets [56,64,65]. Along these lines, pharmacologic inhibition of MTP might also be a potential antiviral strategy for HCV [66]. Several MTP inhibitors [67,68] have already been tested in clinical trials because of their ability to block VLDL secretion. Long-term treatment with MTP inhibitors was associated with elevated liver aminotransferase levels and hepatic fat accumulation [67,68], thus hampering the approval of these drugs for the treatment of hypercholesterolemia on a long-term basis. Shorter treatment regimens reduced the plasma level of VLDL with only minor adverse effects, which disappeared after drug removal [68]. It might be of interest to assess safety and efficacy of short-term treatment with MTP inhibitors in treating HCV infection.

## Conclusions

Understanding the mechanisms underlying liver steatosis in HCV infected patients is currently a focus of great interest and investigations. These studies might greatly contribute to improved clinical management of this frequent infection. In this regard, many questions remain unanswered and there is the need to further investigate the precise mechanisms and complex network of pathways by which HCV and its proteins interfere with hepatic lipid metabolism, particularly with the machinery of assembly and secretion of VLDL.

## Abbreviations used

CHC: chronic hepatitis C; HCV: hepatitis C virus; NAFLD: non-alcoholic fatty liver disease; MTP: microsomal triglyceride transfer protein; BMI: body mass index; LVP: lipo-viral particles; LDL: low density lipoproteins; VLDL: very low density lipoproteins; apoB: apolipoprotein B; apoE: apolipoprotein E.

## Competing interests

The authors declare that they have no competing interests.

## Authors' contributions

SM and AA conceived of the study, and participated in its design and coordination. MH and DB revised and expand the contents of the manuscript. All authors read and approved the final manuscript.
